# Impact of a purported nootropic supplementation on measures of mood, stress, and marksmanship performance in U.S. active duty soldiers

**DOI:** 10.1186/s12970-018-0229-8

**Published:** 2018-05-31

**Authors:** Nicholas Barringer, Aaron Crombie, Russ Kotwal

**Affiliations:** 1 Military Nutrition Division, U.S. Army Research Institute of Environmental Medicine, 10 General Greene Ave, Natick, MA 01760 USA; 2 U.S. Military-Baylor University Graduate Program in Nutrition, AMEDDC&S HRCoE, 3630 Stanley Rd, Bldg 2841, Suite 0308. Joint Base San Antonio-Fort Sam Houston, San Antonio, TX 78234 USA; 3 Department of Defense Joint Trauma System, 3698 Chambers Road, Joint Base San Antonio-Fort Sam Houston, San Antonio, TX 78234 USA

**Keywords:** Marksmanship, Nootropic, Military performance

## Abstract

**Background:**

The purpose of this study was to determine the impact of a commercially available purported nootropic supplement on mood, stress, and rifle marksmanship accuracy and engagement time via an Engagement Skills Trainer.

**Methods:**

In this double-blind, placebo-controlled trial, 43 U.S. active duty Soldiers participating in a professional military course were assigned to treatment (*n* = 20; 16 males and 4 females) or placebo (*n* = 23; 15 males and 8 females) based on initial marksmanship score. The study period was 31 days (testing performed on days 1 and 31, supplementation days 2 through 30). Participants were instructed to consume 2 pills at breakfast and 1 pill at dinner for a total of 3 pills per day (1925 mg) of either the Alpha Brain® nootropic supplement or a placebo. Height, weight, cortisol (in a hair sample), body composition using multi-frequency tetrapolar bioelectrical impedance (InBody 720), and marksmanship (Engagement Skills Trainer 2000). Marksmanship was assessed in the prone position with zeroed M-4 rifles with a twenty target protocol with targets presenting and remaining for 3 s at set intervals. Participants’ performance were assessed with hits versus misses, distance of hit from target center mass (DCM), and target engagement speed. Statistical analysis via SPSS Statistics 21 (IBM) was conducted using a repeated measures ANOVA with significance set at *P* < 0.5.

**Results:**

There was no statistically significant difference between Treatment and Placebo for hits (TreatmentPre 18.5 ± 1.5, TreatmentPost 19.4 ± 0.8, PlaceboPre18.2 ± 2.9, PlaceboPost19.4 ± 1.3), initial reaction time in seconds (TreatmentPre 1.65 ± 0.28, TreatmentPost 1.43 ± 0.28, PlaceboPre1.59 ± 0.29, PlaceboPost1.41 ± 0.21), mean reaction time in seconds (TreatmentPre 1.60 ± 0.20, TreatmentPost 1.41 ± 0.16, PlaceboPre1.61 ± 0.51, PlaceboPost1.46 ± 0.56), or distance from center mass in centimeters (TreatmentPre 11.28 ± 4.28, TreatmentPost 11.92 ± 4.23, PlaceboPre10.52 ± 5.29, PlaceboPost10.94 ± 4.64). A significant time effect (*P* < 0.5) was found for both groups on all variables except distance from center mass. Reaction time values were adjusted to give percent decrease for initial reaction and mean reaction for the Treatment group (− 12.3% ± 16, − 15.2% ± 21.6) compared to the Placebo group (− 8.3% ± 21.8, − 12.5% ± 23.5), but no significant difference was found.

**Conclusion:**

The Alpha Brain® nootropic supplement did not have any statistically significant effects on marksmanship performance in this study. Given the rising popularity of nootropic supplements, future research on their potential impact on cognitively demanding soldier tasks, such as target discrimination scenarios, are recommended.

## Background

Military personnel participate in intense training under extreme physical, mental, and environmental conditions. Trainees are required to perform cognitively demanding tasks while sleep-deprived, in an energy deficit, physically exhausted, and while operating under load. Nootropic supplements offer the potential to optimize cognitive performance and therefore ameliorate cognitive decline associated with environmental and other stressors.

Given research using Alpha Brain® demonstrating improved processing speed and memory [1], we examined whether a nootropic supplement would improve performance of a tactically-relevant cognitive task such as marksmanship. Several Alpha Brain® ingredients have been reported to independently enhance physical and cognitive performance. For example, vitamin B-6, one of the key ingredients in Alpha Brain®, at higher intakes have been associated with better cognitive function as one ages [2] and has been previously reported by Beals et al. that 46% of female and 45% of male Soldiers fall below the Military Dietary Recommended Intake (MDRI) based on self-reported dietary recall [3]. Supplementation with phosphatidylserine, another key ingredient in Alpha Brain®, has been reported to improve cognitive performance in healthy college age males [4]. Phosphatidylserine also demonstrated effectiveness in golf which, like marksmanship, requires the combination of aiming and upper activity while holding an implement [5]. *Bacopa monnieri* (BM), one of the ingredients in the nootropic tested, has demonstrated significant potential to act as neuroprotective anti-oxidant in previous research [6]. The ingredient Uncaria tomentosa was shown to significantly ameliorate the impact of an amnesiac drug in an animal model, and identified as a potential treatment for dysfunction of cholinergic systems in the brain [7]. Similarly, Vipocentine, another active ingredient, has significantly demonstrated the same anti-amnesiac effect in animal models [8]. The ingredient Pterostilbene was effective in combatting mitochondrial dysfunction and oxidative damage associated with cognitive decline in an aging animal models [9]. In addition to showing antioxidant properties, acute ingestion of another component of Alpha Brain, L-alpha-glycerphosporycholine (Alpha GPC), significantly prevented exercise-associated reaction time decrements when compared to a placebo treatment [10]. Tyrosine treatment, another key ingredient, has demonstrated the potential to ameliorate decrements in cognitive function due to physiological stress [11]. For example, Mahoney and colleagues reported tyrosine treatment compared to a placebo prevented declines in cognitive function from cold water exposure [11]. In addition, the authors observed that marksmanship, assessed by average distance from center mass and tightness of shot group, was not impacted in the tyrosine group but it was in the placebo group [11]. Based on these previous reports, it was determined the potential impacts of Alpha Brain® on marksmanship should be examined. The main purpose of this study was to determine if a commercially available nootropic would improve marksmanship performance, specifically accuracy and target acquisition time. Additionally, changes in hair cortisol levels, mood as indicated by Profile of Moods States (POMs) and resiliency as assessed by Dispositional Resilience Scale (DRS-15) results were measured. This study of the dietary supplement, Alpha Brain®, in this research is not intended for therapeutic purposes.

## Methods

Forty-three active duty U.S. Army Soldiers volunteered to participate and were assigned to a treatment (EXP; *n* = 20; 16 males & 4 females) or placebo (PLC; *n* = 23; 15 males and 8 females) groups based on initial marksmanship score. Inclusion Criteria for the study were generally healthy, 25–35 years old, active duty U.S. Soldier, and no underlying medical conditions. Potential participants were excluded from the study if they were currently consuming or had previously consumed a nootropic supplement in the preceding month; had a known cardiovascular, metabolic, psychological, neurological, or pulmonary disease; currently were taking or had taken any anti-coagulate, psychoactive, or Attention Deficit Hyperactivity Disorder medications within the prior month, and/or have been told by a physician to abstain from vigorous activities. Soldiers were provided a pill bottle labeled either A or B and instructed to consume 2 pills with breakfast and 1 pill at dinner for the 30 day study. Study eligibility was based on participant self-report. No medical records were viewed for the purposes of this research. Participants from the Investigators’ chain of command were not permitted and potential participants’ superiors were not present during recruitment briefs to prevent the perception of any command influence.

### Engagement skills trainer (EST)

The EST is a standardized marksmanship simulator that has been demonstrated to have a strong positive correlation to actual marksmanship performance [12]. Participants’ marksmanship performance was assessed utilizing a 20-shot standard course of fire from the prone supported position (lying flat on the ground with weapon supported on sandbags), with targets presented at varying distances. Participants’ accuracy was assessed by hits versus misses, and distance of hits from the target’s center of mass, as well as target acquisition and engagement time (time of target presentation minus shot fired). Initial reaction time was calculated from time first target was presented minus when the volunteer fired.

### Profile of mood states 2 (POMS)

Mood changes were assessed using the POMS 2®. The POMS 2® is a validated [13], standardized, self-rating scale consisting of 65 questions that measures six identifiable mood states: Tension-Anxiety, Depression-Dejection, Anger-Hostility, Vigor-Activity, Fatigue-Inertia, and Confusion-Bewilderment. From these six mood states a Total Mood Disturbance score is calculated.

### Dispositional resilience scale (DRS-15)

The DRS-15 is a short, 15-item hardiness scale that assesses resiliency, and has demonstrated internal consistency, validity and reliability for military academy cadets [14].

### Hair cortisol

Hair cortisol has been used in previous research as a biomarker of stress exposure [15] and previously associated with PTSD in military personnel [16] . Cortisol measured from the blood, urine, or saliva is reflective of a subject’s recent environment; whereas cortisol measured from the hair is reflective of an individual’s long term environment. To assess cortisol levels, hair samples were obtained from participants’ scalp, and samples were analyzed by Viaguard labs in Ontario, Canada, where hair samples were processed using techniques previously established by Sauve et al. [15]

### Body composition

Body composition was assessed utilizing an Inbody 720 through multi-frequency tetrapolar bioelectrical impedance. The Inbody 720 has been shown to be a valid and accurate estimate of body composition [17].

All measures were performed at baseline and 30 days following supplementation. Data were analyzed using IBM SPSS Statistics for Windows (version 22.0; IBM Corp., Armonk, NY), using a repeated measures ANOVA with significance set a priori at *P* < 0.5. All data are presented ±SD.

## Results

### Participants

Compliance was 94% for Treatment and 84% for Placebo. No adverse events were reported or observed for any of the participants involved in the study (Table 1).

**Table 1:** Participant characteristics

	Placebo Group				Treatment Group			
	Pre		Post		Pre		Post	
	Males	Females	Males	Females	Males	Females	Males	Females
Variable	*N* = 15	*N* = 8	*N* = 15	*N* = 8	*N* = 16	*N* = 4	*N* = 16	*N* = 4
Age, years	30.1 ± 2.4	29.1 ± 3.2	30.1 ± 2.4	29.1 ± 3.2	30.8 ± 2.7	31.3 ± 2.9	30.8 ± 2.7	31.3 ± 2.9
Height, cm^a^	179.4 ± 6.3	161.8 ± 6.8	179.4 ± 6.4	161.8 ± 6.8	176.1 ± 6.8	156 ± 9.4	176.1 ± 6.9	156 ± 9.4
Weight, kg^b^	87.9 ± 8.7	64.5 ± 11.9	87.3 ± 8.5	65.7 ± 11.9	81.9 ± 9.9	53.43 ± 9.1	82 ± 9.8	53.9 ± 9.6
BMI^c^ (kg/m^2^)	27.3 ± 2.4	24.6 ± 3.5	27.1 ± 2.6	25.1 ± 3.5	26.4 ± 2.1	22.0 ± 1.3	26.4 ± 2.1	22.1 ± 1.5
FFM, kg	72 ± 6.7	45.9 ± 5.6	71.65 ± 6.7	46.3 ± 5.2	66.8 ± 9.5	39.7 ± 5.6	66.9 ± 9.8	40 ± 6.1
FM, kg	15.9 ± 4.5	18.6 ± 7.5	15.65 ± 4.9	19.4 ± 8.0	15.1 ± 5.0	13.7 ± 4.0	15.1 ± 4.9	31.8 ± 3.7
Bodyfat, %	17.98 ± 4.5	28.36 ± 6.9	17.78 ± 4.5	28.51 ± 7.6	19.36 ± 4.7	25.35 ± 4.0	19.27 ± 4.9	25.6 ± 2.9

Data are presented as mean ± standard deviation. ^a^kg = kilograms, ^b^cm = centimeters, ^c^BMI = body mass index

The only significant demographic variable difference between groups was for mean female weight between Treatment and Placebo (*p* = 0.00). Otherwise, there were no significant changes in bodyweight or body composition for either group from pre- to post-test.

### Marksmanship

**Fig. 1:**
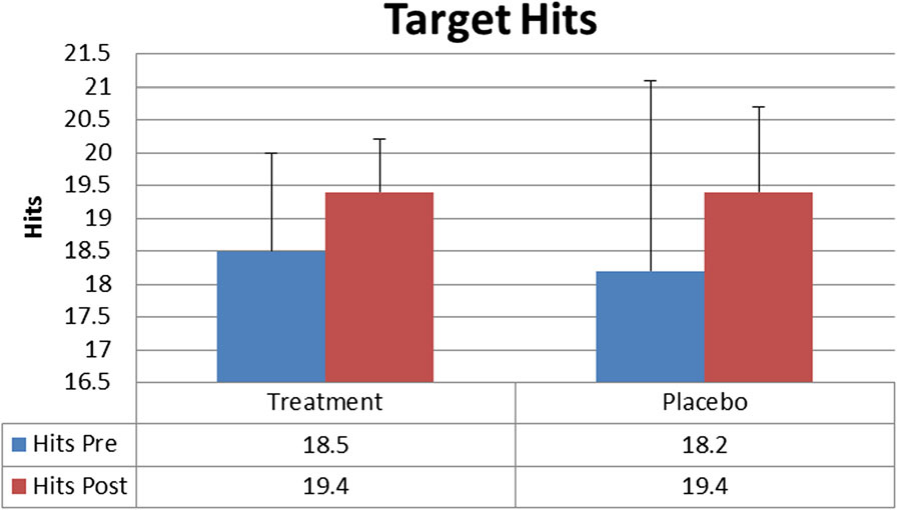
Target Hits

**Fig. 2:**
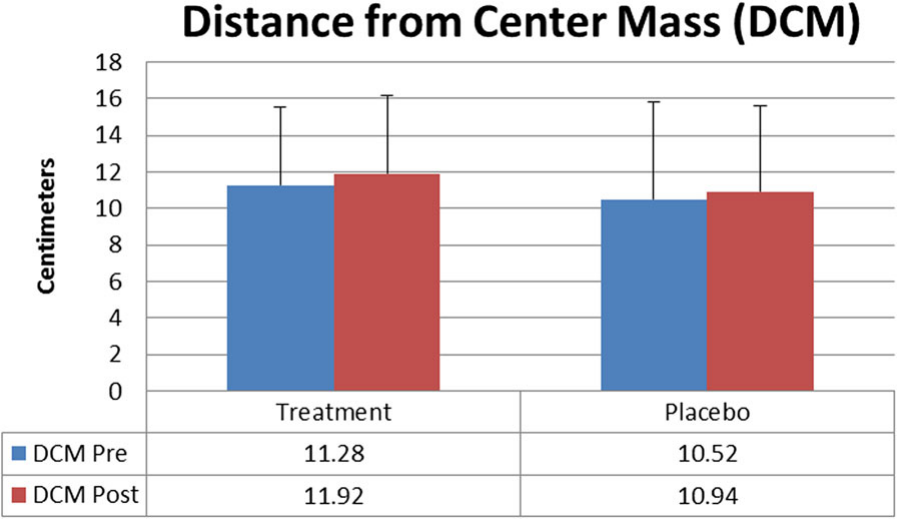
Distance from Center Mass

**Fig. 3:**
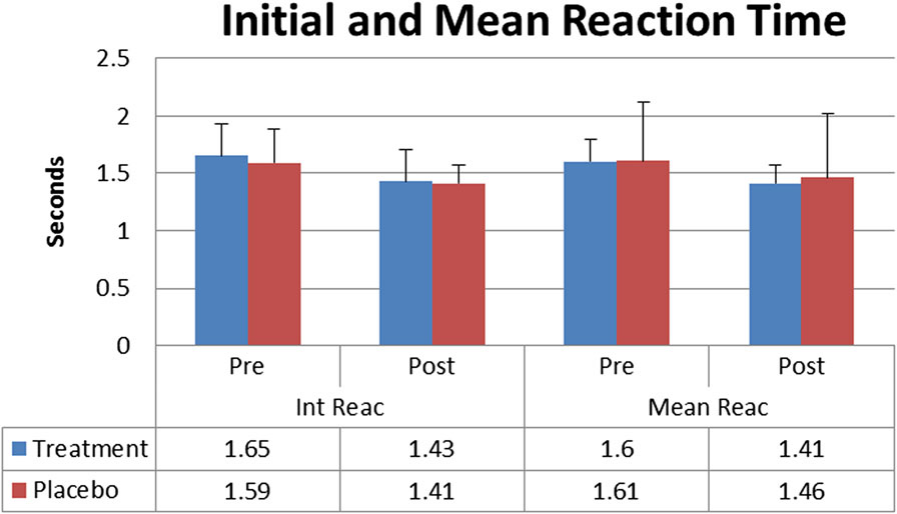
Initial and Mean Reaction Time

There was no statistically significant difference between Treatment and Placebo for hits (*p* = 0.69) (TreatmentPre 18.5 ± 1.5, TreatmentPost 19.4 ± 0.8, PlaceboPre 18.2 ± 2.9, PlaceboPost 19.4 ± 1.3) initial reaction time in seconds (*p* = 0.65) (TreatmentPre 1.65 ± 0.28, TreatmentPost 1.43 ± 0.28, PlaceboPre 1.59 ± 0.29, PlaceboPost 1.41 ± 0.21), mean reaction time in seconds (*p* = 0.52) (TreatmentPre 1.60 ± 0.20, TreatmentPost 1.41 ± 0.16, PlaceboPre 1.61 ± 0.51, PlaceboPost 1.46 ± 0.56), or distance from center mass in centimeters(*p* = 0.87) (TreatmentPre 11.28 ± 4.28, TreatmentPost 11.92 ± 4.23, PlaceboPre 10.52 ± 5.29, PlaceboPost 10.94 ± 4.64). A significant time effect (*P* = 0.00) was found for both groups for all variables except distance from center of mass (*P* = 0.45) demonstrating a potential learning effect of repeating the task. Values for reaction time were adjusted to give percent decrease for initial reaction and mean reaction for the Treatment group (− 12.3% ± 16, − 15.2% ± 21.6) compared to the Placebo group (− 8.3% ± 21.8, − 12.5% ± 23.5) and tested via an independent T-test but no significant difference was found for initial reaction (*p* = 0.52) or mean reaction time (*p* = 0.70) (Figs. 1, 2 and 3).

### POMS2

There was no statistically significant difference for the POMS 2 total mood scale (*p* = 0.47) or in POMS subscale measures (Table 2).

**Table 2:** Profile of Mood States

	Placebo Group		Treatment Group	
	Pre	Post	Pre	Post
Variable	*N* = 23	*N* = 23	*N* = 20	*N* = 20
Depression/Rejection	42.2 ± 3.7	41.6 ± 3.1	43.6 ± 5.6	42.9 ± 5.8
Anger/Hostility	40.6 ± 2.3	40.2 ± 7.2	42.0 ± 4.7	41.8 ± 5.34
Confusion/Bewilderment	42.2 ± 8.1	40.2 ± 7.2	43.0 ± 6.2	40.3 ± 5.8
Fatigue/Inertia	42.9 ± 8.3	41.8 ± 7.8	42.9 ± 8.3	43.3 ± 8.6
Tension/Anxiety	39.9 ± 6.0	39.9 ± 6.9	42.1 ± 6.9	40.1 ± 5.6
Vigor/Activity	52.2 ± 9.1	53.4 ± 10.6	49.5 ± 9.3	49.5 ± 9.5
Total Mood Disturbance	42.9 ± 7.0	41.4 ± 6.98	45.1 ± 7.3	43.7 ± 7.1
Friendliness	51.7 ± 9.4	51.5 ± 10.4	48.6 ± 9.1	49.2 ± 8.9

Data are presented as mean ± standard deviation

### Hair cortisol

Hair cortisol values were similar between groups both before and after the dietary supplement intervention with no change in value over time (*p* = 0.22) (TreatmentPre 44.79 ± 13.23, TreatmentPost 44.5 ± 13.09, and PlaceboPre 43.05 ± 11.61, PlaceboPost 41.53A ± 10.64).

### DRS-15

There were no significant differences for Total Resiliency (*p* = 0.99) scores for either group (Table 3).

**Table 3:** Disposition Resiliency Scale-15

	Placebo Group		Treatment Group	
	Pre	Post	Pre	Post
Variable	*N* = 23	*N* = 23	*N* = 20	*N* = 20
Commitment	5.9 ± 2.1	6.1 ± 2.6	5.5 ± 1.8	5.8 ± 1.6
Control	9.2 ± 2.2	9.6 ± 2.5	9.7 ± 2.4	9.8 ± 2.0
Challenge	−0.2 ± 2.5	−0.2 ± 2.7	−0.6 ± 2.1	−0.6 ± 2.1
Total Resiliency	15.4 ± 5.7	15.8 ± 6.9	14.6 ± 4.5	15.0 ± 4.7

Data are presented as mean ± standard deviation

## Discussion

Nootropic dietary supplements are growing in popularity, but efficacy remains unclear. In this study, we found that 30 days of Alpha Brain® nootropic supplement consumption did not have any statistically significant effects on measures of marksmanship performance, mood, or stress. Despite following a dosing pattern (3 times daily) and daily amount (1972.5 g) consistent with investigations suggesting potential ergogenic benefit [1, 18] we did not see improvement in the number of targets hit, distance from center of mass, or reaction time during a prone supported marksmanship task, in POMS or resiliency scores, or hair cortisol concentrations in rested, otherwise healthy Soldiers.

Our findings are in contrast with two other research studies examining Alpha Brain® efficacy. Solomon et al. [1] reported a significant improvement in verbal memory in healthy young adults after a 6 weeks supplementation at dose consistent with the current study [1].Another study of healthy young adults observed a benefit of the supplement on Event-Related Potential and Electroencephalograph measure of cognitive function after 8 weeks of supplementation [18].

Thus, longer chronic supplementation may have been needed to elicit change.

An area of weakness in both our study and previous studies is a lack of biochemical evidence to support the absorption of the active ingredients in the product. Future research should involve blood samples verifying Alpha Brian’s ingredient absorption rates. A more difficult or variable performance tasks should also be considered. In this study, we studied marksmanship in the prone supported position, which greatly simplifies the task of aligning and accurately firing the weapon. The participants successfully hit 95% of the targets presented. Therefore, a marksmanship task with more upper gain in performance score might be necessary to detect significant improvements, if they are present.

Future investigations examining the efficacy of Alpha Brain® or a similar supplement should consider examining efficacy under more stressful situations. Marksmanship abilities in a relative stress free setting may be mostly predicated on skill. Previous research has demonstrated that external stress, such as load carriage and cold exposure, have demonstrated negative effects on marksmanship that can be sometimes ameliorated by nutritional interventions [11, 19]. Such is the case with one of Alpha Brain’s active ingredients, tyrosine, which was previously shown to be beneficial in promoting cognitive function immediately after a physiological stress of cold water immersion [11]. Similarly caffeine, which has been comprehensively investigated as a cognitive enhancer, demonstrated a greater benefit in marksmanship performance in a military population when they were sleep deprived such as in a sustained operation [20–22].

The military interest in a “Metabolically Optimized Brain” [23] supports the exploration of novel nutritional interventions to cognitively enhance warfighter performance. However, when proposing any form of enhancement within military personnel, ethical concerns [24, 25] should always be weighed and considered. One of the considerations mentioned by Russo concerning the ethical use of pharmacologic fatigue countermeasures is “Have available non-pharmacologic alternatives been fully utilized?” [26] A nutritional nootropic might be such an alternative. Given the rising popularity of nootropic supplements, future research on the potential impact they have on cognitively demanding soldier tasks, such as target discrimination scenarios, should be explored.

## Conclusions

30 days of dosing with the Alpha Brain supplement at 3 pills per or 1972.5 mg per day dosing had no appreciable effect on marksmanship, mood or cortisol levels in otherwise well rested Soldiers engaged in a basic marksmanship test.

## References

[1] Solomon TM, Leech J, Murphy C, DeBros G, Budson A, Solomon P (2015). A randomized, double-blind, placebo controlled, parallel group, efficacy study of alpha BRAIN® administered orally. Journal of the International Society of Sports Nutrition.

[2] Qin B, Xun P, Jacobs Jr., DR, Zhu N, Daviglus ML, Reis JP, He K. Intake of niacin, folate, vitamin B-6, and vitamin B-12 through young adulthood and cognitive function in midlife: the coronary artery risk development in young adults (CARDIA) study. Am J Clin Nutr. 2017;106(4):1032–40. 10.3945/ajcn.117.157834 PMC5611785 28768650

[3] Beals K, Darnell ME, Lovalekar M (2015). Suboptimal nutritional characteristics in male and female soldiers compared to sports nutrition guidelines. Mil Med.

[4] Parker AG, Byars A, Purpura M, Jäger R (2015). The effects of alpha-glycerylphosphorylcholine, caffeine or placebo on markers of mood, cognitive function, power, speed, and agility. Journal of the International Society of Sports Nutrition..

[5] Jäger R, Purpura M, Geiss K-R (2007). The effect of phosphatidylserine on golf performance. Journal of the International Society of Sports Nutrition..

[6] Aguiar S, Borowski T (2013). Neuropharmacological review of the nootropic herb Bacopa monnieri. Rejuvenation Res.

[7] Mohamed A, Matsumoto K, Tabata K, Takayama H, Kitajima M, Watanabe H (2000). Effects of Uncaria tomentosa total alkaloid and its components on experimental amnesia in mice: elucidation using the passive avoidance test. J Pharm Pharmacol.

[8] Shang Y, Wang L, Li Y, P-f G (2016). Vinpocetine improves scopolamine induced learning and memory dysfunction in C57 BL/6J mice. Biol Pharm Bull.

[9] Naik B, Nirwane A, Majumdar A (2017). Pterostilbene ameliorates intracerebroventricular streptozotocin induced memory decline in rats. Cogn Neurodyn.

[10] Hoffman JR, Ratamess NA, Gonzalez A (2010). The effects of acute and prolonged CRAM supplementation on reaction time and subjective measures of focus and alertness in healthy college students. Journal of the International Society of Sports Nutrition..

[11] Mahoney CR, Castellani J, Kramer FM, Young A, Lieberman HR (2007). Tyrosine supplementation mitigates working memory decrements during cold exposure. Physiol Behav.

[12] Hagman JD (1998). Using the engagement skills trainer to predict rifle marksmanship performance. Mil Psychol.

[13] Nyenhuis DL, Yamamoto C, Luchetta T, Terrien A, Parmentier A (1999). Adult and geriatric normative data and validation of the profile of mood states. J Clin Psychol.

[14] Bartone PT (2007). Test-retest reliability of the dispositional resilience scale-15, a brief hardiness scale. Psychol Rep.

[15] Sauvé B, Koren G, Walsh G, Tokmakejian S, Van Uum SH (2007). Measurement of cortisol in human hair as a biomarker of systemic exposure. Clinical & Investigative Medicine.

[16] Steudte-Schmiedgen S, Stalder T, Schönfeld S (2015). Hair cortisol concentrations and cortisol stress reactivity predict PTSD symptom increase after trauma exposure during military deployment. Psychoneuroendocrinology.

[17] Ogawa H, Fujitani K, Tsujinaka T (2011). InBody 720 as a new method of evaluating visceral obesity. Hepato-Gastroenterology.

[18] Leech JD, Cecchi M, Solomon TM, Solomon PR (2015). Effects of the nootropic compound alpha Brain® on ERP and EEG measures of cognitive performance. Alzheimer's & Dementia: The Journal of the Alzheimer's Association.

[19] Tharion WJ, Moore RJ (1993). Effects of carbohydrate intake and load bearing exercise on rifle marksmanship performance.

[20] McLellan TM, Bell DG, Lieberman HR, Kamimori GH (2003). The impact of caffeine on cognitive and physical performance and marksmanship during sustained operations.

[21] Lieberman HR, Tharion WJ, Shukitt-Hale B, Speckman KL, Tulley R (2002). Effects of caffeine, sleep loss, and stress on cognitive performance and mood during US navy SEAL training. Psychopharmacology.

[22] Tharion WJ, Shukitt-Hale B, Lieberman HR (2003). Caffeine effects on marksmanship during high-stress military training with 72 hour sleep deprivation. Aviat Space Environ Med.

[23] Elfenbaum P, Crawford C, Enslein V, Berry K (2017). Priorities for implementing nutritional science into practice to optimize military performance. Nutr Rev.

[24] Shunk D (2015). Ethics and the enhanced soldier of the near future. Mil Rev.

[25] Michaud-Shields M. Personal augmentation–the ethics and operational considerations of personal augmentation in military operations. Canadian Military Journal. 2014;15(1)

[26] Russo MB (2007). Recommendations for the ethical use of pharmacologic fatigue countermeasures in the US military. Aviat Space Environ Med.

